# MicroRNA-21-3p accelerates diabetic wound healing in mice by downregulating SPRY1

**DOI:** 10.18632/aging.103610

**Published:** 2020-07-07

**Authors:** Yaohong Wu, Kun Zhang, Rong Liu, Hexing Zhang, Dong Chen, Shuangqi Yu, Wei Chen, Song Wan, Yi Zhang, Zhiwei Jia, Rongchun Chen, Fan Ding

**Affiliations:** 1 Department of Orthopedics, The Affiliated Ganzhou Hospital of Nanchang University, Ganzhou 341000, Jiangxi, China; 2 Department of Orthopedics, Wuhan Fourth Hospital, Puai Hospital, Tongji Medical College, Huazhong University of Science and Technology, Wuhan 430033, Hubei, China; 3 Department of Orthopaedics, Puren Hospital, Wuhan University of Science and Technology, Wuhan 430081, Hubei, China; 4 Department of Orthopedics, Dongzhimen Hospital, Beijing University of Chinese Medicine, Beijing 100700, China

**Keywords:** microRNA-21-3p, diabetes, wound healing, SPRY1

## Abstract

A variety of novel drugs and advanced therapeutic strategies have been developed for diabetic foot ulcers (DFUs); however, the clinical outcomes are unsatisfactory and the underlying mechanisms of DFU remain elusive. MicroRNAs (miRNA) regulate the pathological processes of many diseases. Fibroblasts are involved in each stage of wound healing, and the functions of fibroblasts may be regulated by miRNAs. In the present study, we found that the levels of miRNA-21-3p (miR-21-3p) were decreased in patients with diabetes as compared with those in the healthy control. Similarly, the level of miRNA-21-3p was decreased in fibroblasts that were stimulated with D-glucose as compared with that in the control fibroblasts. Furthermore, enhanced function was found in fibroblasts followed by the miR-21-3p agonist treatment, and a rapid wound healing process was achieved in the miR-21-3p agonist-treated mice. MiR-21-3p directly targeted protein sprout homolog 1 (SPRY1), and the miR-21-3p-regulated reduction in SPRY1 enhanced the function of fibroblasts and accelerated wound healing *in vivo*. These findings suggest that miR-21-3p may treat DFU by reducing SPRY1.

## INTRODUCTION

Diabetic foot ulcers (DFUs) are serious complications of diabetes that often occur in elderly people and seriously affect their daily lives. Since the incidence of DFU is increasing because of the developing aging population [1–2], many advanced drugs and therapies have been designed to treat DFU. For example, a novel bio-active cupriferous hollow nanoshell was developed as a photothermal therapeutic agent for the treatment of diabetic wounds and achieved satisfactory outcomes *in vitro* [3]. Similarly, a nanozyme-reinforced self-protecting hydrogel promoted angiogenesis in diabetic wounds [4]. However, the clinical outcomes of patients with DFUs are unsatisfactory, and the mechanisms underlying this complication remain elusive.

Fibroblasts play vital roles in each stage of wound healing, and the proper functioning of fibroblasts is closely associated with improved wound healing [5]. MicroRNAs (miRNAs) are small noncoding single-stranded RNAs that regulate gene expression *in vivo*. Results from previous studies have revealed that fibroblasts may be regulated by miRNAs in multiple diseases [6–7]. For example, Qin et al. [8] reported that miR-17-5p regulated the function of fibroblasts to enhance the progression of heterotopic ossification. Another study demonstrated that miR-494 promoted the proliferation, migration, and invasion of fibroblasts to regulate myocardial infarction [9].

Sprouty homolog 1 (SPRY1) acts as an antagonist of fibroblast growth factor (FGF) pathways and may negatively modulate respiratory organogenesis [10]. Additionally, studies have reported that SPRY1 was expressed in fibroblasts and affected several signaling cascades that regulate renal fibrosis [11]. A recent study also demonstrated that the inhibition of SPRY1 played a vital role in successful wound repair [12].

Thus, the current study aimed to identify the miRNAs that were differentially expressed in patients with diabetes and healthy controls and explored how these miRNAs regulated the functions of fibroblasts and wound healing. Our results showed that the expression of SPRY1 was significantly down-regulated by miR-21-3p *in vitro* and *in vivo*. Therefore, SPRY1 may target miR-21-3p to induce diabetic wound healing.

## RESULTS

### MiR-21-3p expression in fibroblasts was suppressed following glucose stimulation

**Figure 1:**
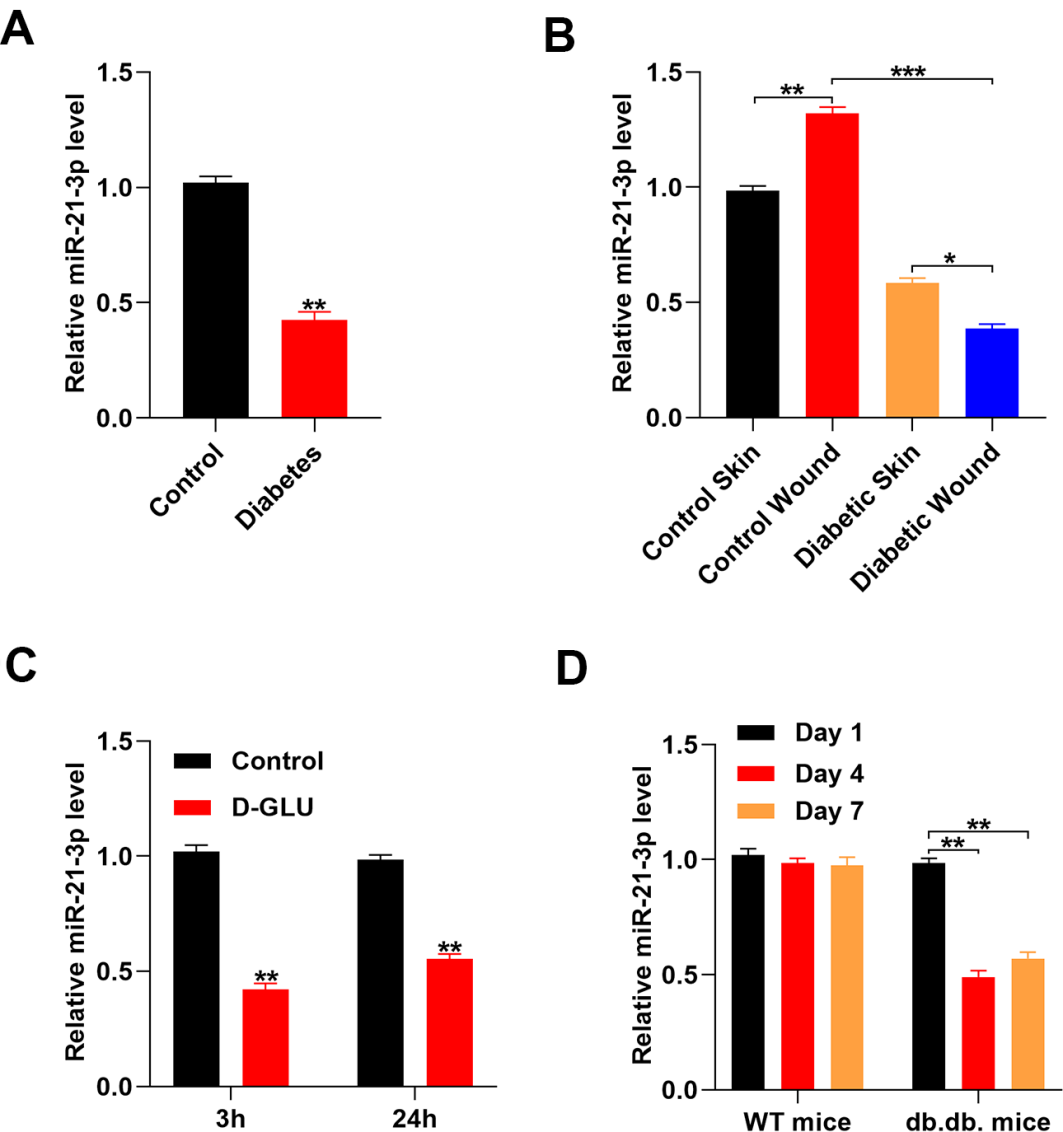
**Glucose stimulation suppresses miR-21-3p expression.** (**A**) The level of miR-21-3p in the diabetes patients and the healthy controls was measured by qRT-PCR analysis (n=10, per group); (**B**) qRT-PCR was used to measure MiR-21-3p expression in the skin tissue from mice that received different treatments (n=10, per group); (**C**) The level of miR-21-3p in fibroblasts was measured at 3 h and 24 h following diabetic stimulation with D-glucose; (**D**) The level of miR-21-3p in WT and db.db. mice tissues was measured on days 1, 4, and 7 post-wounding. (n=10, per group). Data are presented as the mean ± SD from three independent experiments. *p < 0.05, **p < 0.01, ***p < 0.001.

A previous study reported that exosomal miR-21-3p that was derived from human umbilical cord blood was able to promote angiogenesis and fibroblast function [13]. Thus, we explored if the expression of miR-21-3p in fibroblasts was regulated by glucose stimulation and exerted wound healing effects. First, we collected serum samples from patients with diabetes and healthy controls, and the levels of miR-21-3p in these samples were measured by quantitative real-time PCR (qRT-PCR). The results indicated that the level of miR-21-3p was suppressed in the patients with diabetes as compared with that in the healthy volunteers. (Figure 1A). Next, we created a diabetic wound in mice and measured the levels of miR-21-3p in the different mouse groups. The results showed that the level of miR-21-3p was increased after wounding, and the level of miR-21-3p in the diabetic mice was lower than that in the non-diabetic mice (Figure 1B). To determine if miR-21-3p expression in fibroblasts was altered in response to a diabetic stimuli, we measured changes in the level of miR-21-3p in fibroblasts that were treated with 20 mM D-glucose via qRT-PCR analysis. The results from the qRT-PCR analysis revealed that the level of miR-21-3p was decreased in the D-glucose-stimulated fibroblasts as compared with that in the control fibroblasts at 3 h and 24 h post-stimulation (Figure 1C). Similarly, the level of miR-21-3p was significantly decreased at 4 and 7 days post-wounding as compared with that at 1 day post-wounding in the diabetic mice, however, this similar result was not detected in non-diabetic mice (Figure 1D).

### MiR-21-3p enhances fibroblast function *in vitro*

**Figure 2:**
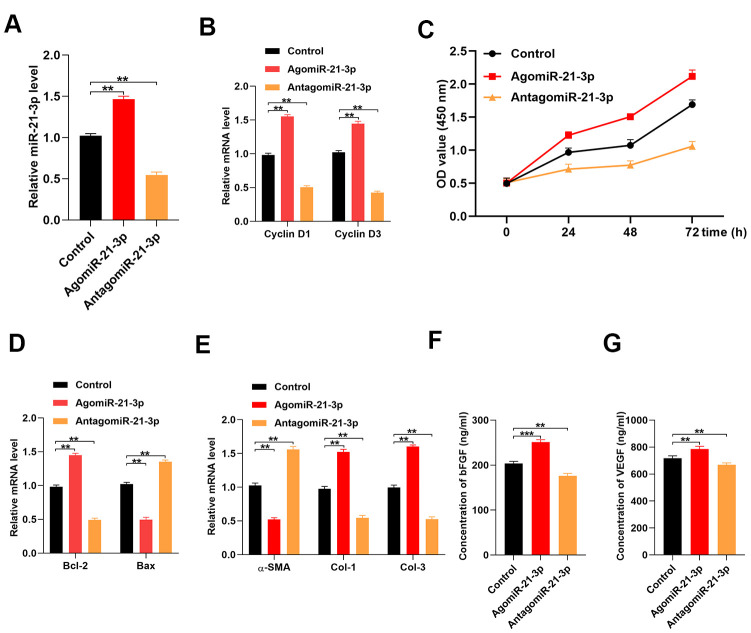
**MiR-21-3p promotes fibroblast function in vitro.** (**A**) The level of miR-21-3p in the control fibroblasts and the fibroblasts that were transfected with agomiR-21-3p or antagomiR-21-3p was measured by qRT-PCR; (**B**) The levels of cyclin D1 and cyclin D3 mRNA in the different groups were measured by qRT-PCR; (**C**) CCK-8 detected the proliferative ability of fibroblasts in the different groups; (**D**) The levels of Bcl-2 and Bax mRNA in the different groups were measured by qRT-PCR; (**E**) The levels of α-SMA, Col 1, and Col 3 mRNA in the different groups were measured by qRT-PCR; (**F**, **G**) The concentrations of bFGF and VEGF were measured by ELISA. Data are presented as the mean ± SD from three independent experiments. *p < 0.05, **p < 0.01, ***p < 0.001.

To investigate the effect of miR-21-3p on fibroblasts function, we designed an miR-21-3p agonist (agomiR-21-3p) and antagonist (antagomiR-21-3p). Results from the qRT-PCR analysis indicated that the level of miR-21-3p was significantly increased in the agomiR-21-3p-treated fibroblasts and significantly decreased in the antagomiR-21-3p-treated fibroblasts as compared with that in the control fibroblasts (Figure 2A). Next, the levels of the cell proliferation-related genes, *cyclin D1* and *cyclin D3*, were detected in the control agomiR-21-3p, and antagomiR-21-3p groups using qRT-PCR analysis. Results from tis assays suggested that the relative mRNA levels of cyclin D1 and cyclin D3 were significantly increased in the agomiR-21-3p-treated fibroblasts and significantly decreased in the antagomiR-21-3p-treated fibroblasts as compared with those in the control fibroblasts (i.e., miR-21-3p enhanced the proliferation of fibroblasts) (Figure 2B. Consistently, results from the cell counting kit-8 (CCK-8) assay demonstrated that miR-21-3p enhanced the proliferation of fibroblasts (Figure 2C). The results from qRT-PCR also revealed that the relative mRNA level of the cell apoptosis-related gene, *Bcl-2*, was significantly increased in the agomiR-21-3p-treated fibroblasts and significantly decreased in the antagomiR-21-3p-treated fibroblasts as compared with that in the control fibroblasts (Figure 2D). Conversely, the relative level of *Bax* was significantly decreased in the agomiR-21-3p-treated fibroblasts and significantly increased in the antagomiR-21-3p-treated fibroblasts as compared with that in the control fibroblasts, indicating that miR-21-3p suppressed the apoptosis of fibroblasts (Figure 2D). Moreover, the impact of the miR-21-3p on soluble collagen synthesis in fibroblasts was also assessed, and the results suggested that the relative mRNA levels of α-SMA, Collagen I (Col 1), and Collagen III (Col 3) were significantly changed in the agomiR-21-3p-treated fibroblasts and significantly decreased in the antagomiR-21-3p-treated fibroblasts as compared with those in the control fibroblasts (Figure 2E). Additionally, the results from the ELISA indicated that the concentrations of basic fibroblast growth factor (bFGF) and vascular endothelial growth factor (VEGF) were higher in the agomiR-21-3p-treated fibroblasts and lower in the antagomiR-21-3p-treated fibroblasts than those in the control fibroblasts (Figure 2F, 2G).

### MiR-21-3p exerts its effect via directly targets SPRY1

**Figure 3:**
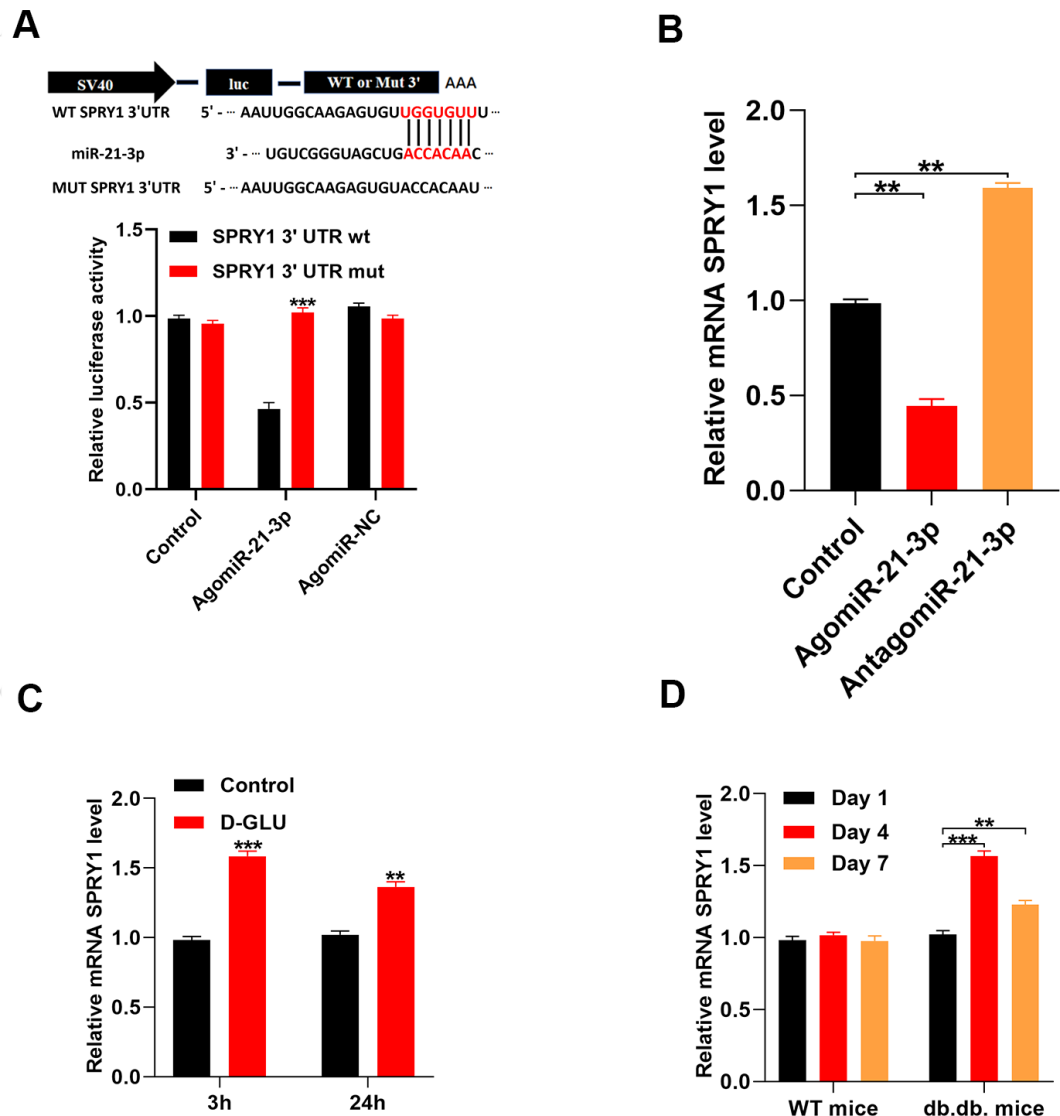
**MiR-21-3p directly targets SPRY1.** (**A**) The luciferase assay results of miR-21-3p and SPRY1; (**B**) The level of miR-21-3p in the different groups was measured by qRT-PCR; (**C**) The level of mRNA SPRY1 in fibroblasts was measured at 3 h and 24 h following diabetic stimulation with D-glucose; (**D**) The level of mRNA SPRY1 in mice tissues was measured on days 1, 4, and 7 post-wounding (n=10, per group). Data are presented as the mean ± SD from three independent experiments. *p < 0.05, **p < 0.01, ***p < 0.001.

Results from previous studies suggest that SPRY1 is closely related to fibroblast function and wound repair [14]. We performed a luciferase reporter assay to determine if miR-21-3p could bind to the predicted target region of the SPRY1 mRNA. When this target region was mutated, the miRNA was no longer able to bind and suppress luciferase activity (Figure 3A). Furthermore, the level of SPRY1 mRNA was markedly decreased in agomiR-21-3p-treated fibroblasts as compared with that in the control fibroblasts (Figure 3B) To investigate whether SPRY1 expression was altered in response to diabetic stimuli in fibroblasts, we examined the level of SPRY1 mRNA in fibroblasts that were treated with 20 mM D-glucose using qRT-PCR analysis. The results revealed that the level of SPRY1 mRNA was significantly increased in the D-glucose-treated fibroblasts as compared with that in the control fibroblasts at 3 h and 24 h post-stimulation, respectively (Figure 3C). Similarly, mRNA SPRY1 expression was significantly increased at 4 and 7 days post-wounding as compared with that at 1 day post-wounding in diabetic mice; however, this result was not detected in non-diabetic mice (Figure 3D).

### Reduction in SPRY1 enhanced fibroblasts function

**Figure 4:**
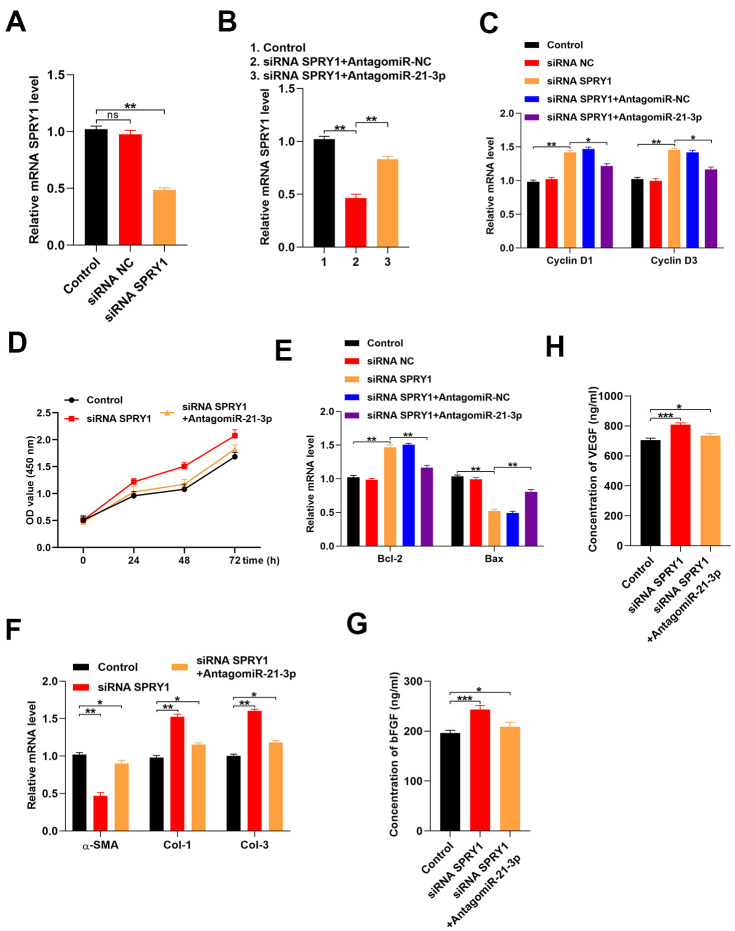
**Reduction of SPRY1 enhances fibroblast function.** (**A**, **B**) The level of mRNA SPRY1 in the different groups was measured by qRT-PCR; (**C**) The levels of cyclin D1 and cyclin D3 mRNA in the different groups were measured by qRT-PCR; (**D**) CCK-8 assay detected the proliferative ability of fibroblasts in the different groups; (**E**) The levels of Bcl-2 and Bax mRNA in the different groups were measured by qRT-PCR; (**F**) The levels of α-SMA, Col 1, and Col 3 mRNA in the different groups were measured by qRT-PCR; (**G**, **H**) The concentrations of bFGF and VEGF were measured by ELISA. Data are presented as the mean ± SD from three independent experiments. *p < 0.05, **p < 0.01, ***p < 0.001.

To determine if fibroblast function was SPRY1-dependent, we transfected fibroblasts with an siRNA that was specific for SPRY1. Our results indicated that the level of SPRY1 was markedly decreased in the siRNA-treated fibroblasts as compared with that in the control fibroblasts (Figure 4A). Additionally, the inhibition of miR-21-3p partially reversed the suppression of SPRY1 mRNA (Figure 4B). To investigate the effect of SPRY1 on the proliferation of fibroblasts, the levels of cyclin D1 and cyclin D3 were detected using the CCK-8 assay. The results indicated that the proliferation of fibroblasts was enhanced in the siRNA-SPRY-treated fibroblasts as compared with that in the control fibroblasts, and the inhibition of miR-21-3p partially reversed this effect (Figure 4C, 4D). Next, the apoptosis-related genes were measured in the fibroblasts, and the result showed that the apoptosis of fibroblasts was inhibited in the siRNA-SPRY1-treated fibroblasts as compared with that in the control fibroblasts, and the inhibition of miR-21-3p partially reversed this effect (Figure 4E). Furthermore, the inhibition of SPRY1 significantly changed the expressions of α-SMA, Col 1, and Col 3 (Figure 4F). Additionally, the results from the ELISA indicated that the concentrations of bFGF and VEGF in the siRNA-SPRY1-treated fibroblasts were higher than those in the control fibroblasts. (Figure 4G, 4H).

### MiR-21-3p accelerates wound healing *in vivo*

**Figure 5:**
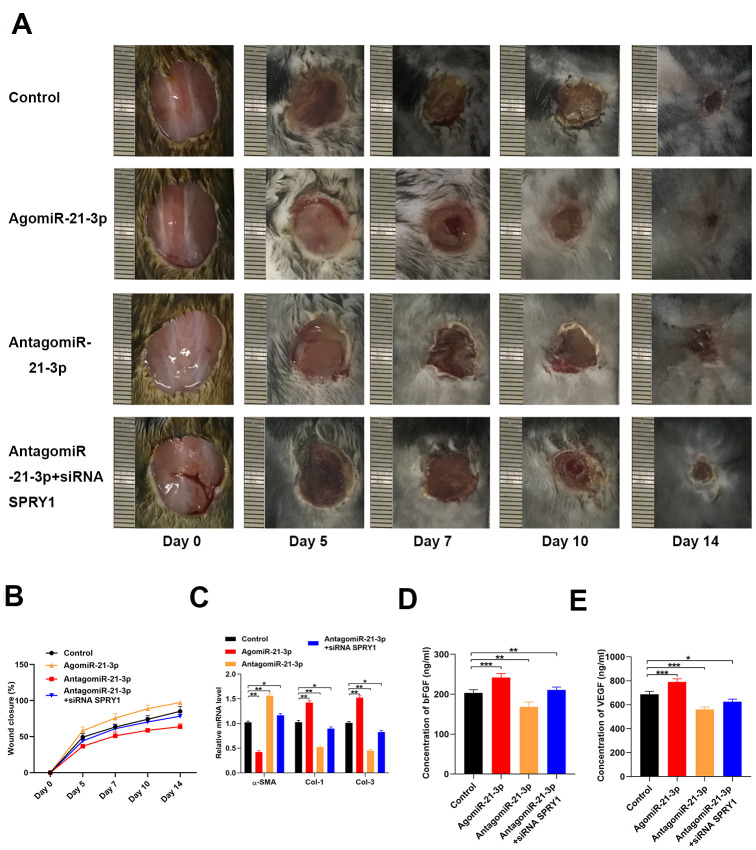
**MiR-21-3p accelerates wound healing *in vivo.*** (**A**) General view of the wound healing process in the mice that received different treatments (n=10, per group); (**B**) The closure rate of the wound in the mice that received different treatments; (**C**) The levels of α-SMA, Col 1, and Col 3 mRNA in the tissues from mice in the different treatment groups were measured by qRT-PCR (**D**, **E**) The concentrations of bFGF and VEGF in the tissues from the different treatment groups were determined by ELISA. Data are presented as the mean ± SD from three independent experiments. *p < 0.05, **p < 0.01, ***p < 0.001.

We investigated if miR-21-3p to promoted wound healing *in vivo* using mice with full-thickness dorsal wounds that were locally injected with either PBS, agomiR-21-3p, antagomiR-21-3p, or antagomiR-21-3p + siRNA-SPRY1 on days 0, 5, 7, 9, and 11 post-wounding. The wound closure rate was significantly higher in the agomiR-21-3p-treated mice (Figure 5A, 5B). Furthermore, wound area skin samples was collected at 14 days post-wounding, and the results from qRT-PCR analyses revealed that the levels of α-SMA, Col 1, and Col 3 were significantly changed in the agomiR-21-3p-treated groups as compared with those in the other groups (Figure 5C). Finally, the results from the ELISA indicated that the amounts of bFGF and VEGF that were secreted from fibroblasts were higher in the agomiR-21-3p-treated mice compared with the control group (Figure 5D, 5E).

## DISCUSSION

The advanced therapy options for DFU have attracted attention from clinical and biochemistry researchers [15]. Results from a recent study revealed that the miR-1248/CITED2/HIF-1α axis played a significant role in diabetic wound healing, indicating that miR-1248 may be used as a novel therapeutic target for wound healing in patients with diabetes [16]. Another study that focused on the role of diabetic stimuli in delayed wound healing in elderly patients suggested that glycation contributed to changes in macrophage activity and cytokine expression. These findings also contribute to our understanding of disturbed wound healing during aging and diabetes [17]. Similarly, in the present research, we aimed to explore the mechanisms that underly the diabetes-associated impaired process of wound healing and identify a novel and feasible target for the treatment of DFU.

MiRNAs are small non-coding RNA molecules that are involved in the pathological processes of various diseases [18–21]. A recent article reported that the lncRNA Rhno1/miR-6979-5p/BMP2 axis was involved in the regulatory mechanism that controls osteoblast differentiation; thus, Rhno1/miR-6979-5p/BMP2 may be a novel therapeutic strategy for fracture healing [22]. Another study reported that miR-7212-5p was associated with N6-methyladenosine modification and played a significant role in fracture healing [23]. Fibroblasts are involved in each stage of wound healing, and the proper functioning of fibroblasts is closely associated with better wound healing. Since large amounts of miRNAs circulate in the blood, fibroblasts are likely to be exposed to these small molecules; therefore, they may be susceptible to the regulatory effects of miRNAs. In the present study, we found that the level of miR-21-3p was lower in diabetic patients than that in healthy controls. Similarly, the level of miR-21-3p was lower in D-glucose- stimulated fibroblasts than that in control fibroblasts. Additionally, the function of fibroblasts was enhanced following treatment with agomiR-21-3p, and a rapid wound healing process was achieved in the miR-21-3p agonist-treated mice. According to these findings, miR-21-3p may regulate the healing process of diabetic wounds; therefore, we aimed to identify the mechanisms underlying miR-21-3p-induced wound healing.

Multiple studies have demonstrated that SPRY1 was involved in the regulation of many diseases. Additionally, SPRY1 was reported to play a selective role in a subset of triple-negative breast cancers to promote the malignant phenotype by enhancing the EGF-mediated mesenchymal phenotype [24]. Moreover, SPRY1 is expressed in fibroblasts and affects several signaling cascades that regulate renal fibrosis and biological process [11, 25]. For example, results from a previous study revealed that the inhibition of SPRY1 promoted wound healing [26]. Thus, in the present research, we hypothesized that SPRY1 was a potential target for miR-21-3p and demonstrated the association between SPRY1 and miR-21-3p using the luciferase assay. We also found SPRY1 expression was significantly down-regulated by miR-21-3p, which is consistent with the *in vitro* results.

Overall, we demonstrated that miR-21-3p was suppressed by diabetic stimuli. Additionally, miR-21-3p positively regulated fibroblast function via reducing SPRY1 and accelerated wound healing *in vivo*. Thus, the overexpression of miR-21-3p may be a novel therapy that achieves satisfactory outcomes for patients with DFU.

## MATERIALS AND METHODS

### Animal experiment

Male db/db mice (weight, 18-20 g) were purchased from Cyagen (https://www.cyagen.com/cn/zh-cn/), and male C57BL/6 mice (6-weeks-old; weight, 18-20 g) were donated from the animal laboratory at the Wuhan University of Science and Technology. To establish the wound model, the mice were initially anesthetized with intraperitoneal injections of sodium pentobarbital (50 mg/kg; Sigma-Aldrich, MO, USA). Next, we created full-thickness cutaneous skin wounds (10 mm diameter) on the dorsum of each mouse. The C57BL/6 mice were randomly divided into the following four groups: 1) Control group (wounds treated with 150 μl PBS), 2) AgomiR-21-3p group (wounds treated with 150 μl of 20 μM AgomiR-21-3p), 3) AntagomiR-21-3p group (wounds treated with 150 μl of 20 μM AntagomiR-20b-5p), and 4) AntagomiR-21-3p + siRNA SPRY1 group (wounds treated with 150 μl of 20 μM AntagomiR-20b-5p and 150 μl of siRNA SPRY1). At post-operative days 0, 5, 7, 10, the wounds were photographed and measured using ImageJ software (National Institutes of Health). The mice were sacrificed at 14 days post-wounding, and skin samples were harvested.

### Blood collection

From July 2017 to August 2019, peripheral blood samples were collected from 10 DUF patients and 10 healthy volunteers at Wuhan Puren Hospital to determine the differences in miRNA levels.

### Fibroblast culture and transfection

Human skin fibroblasts (FuHeng Biology, Shanghai, China) were cultured in high-glucose Dulbecco’s modified eagle medium (Gibco, Grand Island, USA) supplemented with 10% fetal bovine serum and incubated at 37 °C with 5% CO_2_ and 95% humidity. We added 20 mM D-glucose to the fibroblast culture to induce diabetic stimulation in the stimuli group. Next, the fibroblasts were transfected with 200 mM agomiR-21-3p, 200 nM antagomiR-20b-5p (GenePharma, Shanghai), 50 nM miRNA, or siRNA oligos (RIBOBIO, Guanzhou) using Lipofectamine 3000 (ThermoFisher Scientific) according to the manufacturer’s instructions.

### qRT-PCR analysis

Skin samples were preserved using RNA Later (#76104, QIAGEN, Germany), total RNA was isolated from cells using Trizol (Invitrogen, USA), and reverse transcription was performed using the RevertAid First Strand cDNA Synthesis Kit (Thermo Scientific). qRT-PCR was performed using the FastStart Universal SYBR Green Master mix (Rox) (Roche) and 1 μl of reverse transcriptase. GAPDH was used as the reference gene, and the comparative Ct method (2^-ΔΔCt^) was used to calculate fold changes in the expression levels of mRNAs. The sequences of primers were as follows: miR-21-3p: F: 5′-TAGCTTATCAGACTGATG-3′, R: 5′-TGGTGTCGTGGAGTCG-3′; U6: F: 5′-TCCGATCGTGAAGCGTTC-3′, R: 5′-GTGCAGGGTCCGAGGT-3′; SPRY1: F: 5′-ACCCTTCCTGTGTTTTCAT-3′, R: 5′-AGTCACCTTGCTTTTCTTG-3′; Cyclin D1:F: 5′-TTGCCCTCTGTGCCACAGAT-3′, R: 5′-TCAGGTTCAGGCCTTGCACT-3′; Cyclin D3: F: 5′-CTGGCCATGAACTACCTGGA-3′, R: 5′-CCAGCAAATCATGTGCAATC-3′; α-SMA, F: 5′-TGACCCAGATTATGTTTGAGACC-3′, R: 5′-CCAGAGTCCAGCACAATACCA-3′; Col-1: F: 5′-AGGCCACGCATGAGCCGAAG-3′, R: 5′-GCCATGCGTCAGGAGGGCAG-3′; Col-3: F: 5′-AGGATCTGAGGGCTCGCCAGG-3′, R: 5′-AGCCACCAGACTTTTCACCTCCA-3′; GAPDH: F: 5′-CCGTTGAATTTGCCGTGA-3′, R: 5′-TGATGACCCTTTTGGCTCCC-3′.

### ELISA

The concentrations of basic fibroblast growth factor (bFGF) and VEGF in fibroblasts or tissue suspensions were determined using a sandwich ELISA according to the manufacturer’s instructions (enzyme-linked immunosorbent assay kits; Minneapolis, MN, USA). After media collection, the cells in each culture well were counted, and the ELISA values were corrected using the total cell numbers. The concentrations of bFGF and VEGF in each sample were calculated based on the standard curve.

### CCK-8 assay

The proliferation of fibroblasts was determined using a CCK-8. Briefly, the fibroblasts were added to 96-well plates and cultured for 24, 48, or 72 hours, respectively. Next, serum-free medium supplemented with the CCK-8 reagent was added to each group for 2 hours, and the absorbance was measured at 450 nm.

### Luciferase reporter assay

The 3790-3796 position of the 3′ UTR of the SPRY1 mRNA containing the putative target site of miR-21-3p was determined by TargetScan (version 7.0; http://www.targetscan.org/vert_70/), amplified by PCR using cDNA from the fibroblasts, and ligated into the pGL3-basic vector (Promega Corporation). The pGL3-SPRY1-3′ UTR-mutant vector (Mut) was created by introducing two site mutations into the potential miR-21-3p target sites using Quick ChangeSite-Directed Mutagenesis kits (Agilent Technologies, Inc.). The pGL3-SPRY1-3′ UTR-wild-type (W; 200 ng) or pGL3-SPRY1-3′ UTR-Mut (200 ng) vectors were co-infected with the *Renilla* plasmid into fibroblasts using Lipofectamine^®^ 3000 (Thermo Fisher Scientific, Inc.). The Dual-Luciferase Reporter assay system (Promega Corporation) was used to measure the relative luciferase activity in each well. The firefly luciferase expression was normalized to *Renilla*.

### Statistical analysis

All data are displayed as the mean ± SD. Student’s unpaired t-tests were performed to compare difference between two groups. Differences between three or more groups were determined using one-way analysis of variance (ANOVA) and Tukey’s post-hoc analysis when applicable. P < 0.05 was considered statistically significant.

### Ethical statement

Informed consent was completed before this study. Study protocols were approved by Ethic Committee of Wuhan Puren Hospital, Wuhan University of Science and Technology. All experimental animal protocols were collaborated in compliance with the Guide for the Care and Use of Laboratory Animal and approved by the Animal Care Committee of Wuhan Puren Wuhan University of Science and Technology.
